# Biochemistry of the Respiratory Syncytial Virus L Protein Embedding RNA Polymerase and Capping Activities

**DOI:** 10.3390/v15020341

**Published:** 2023-01-25

**Authors:** Priscila Sutto-Ortiz, Jean-François Eléouët, François Ferron, Etienne Decroly

**Affiliations:** 1 Aix Marseille Université, CNRS, AFMB, UMR, 7257 Marseille, France; 2 Unité de Virologie et Immunologie Moléculaires, INRAE, Université Paris Saclay, F78350 Jouy en Josas, France; 3 European Virus Bioinformatics Center, Leutragraben 1, 07743 Jena, Germany

**Keywords:** human respiratory syncytial virus, antiviral, polymerase, capping, methyltransferase, polyribonucleotidyl transferase, drug design

## Abstract

The human respiratory syncytial virus (RSV) is a negative-sense, single-stranded RNA virus. It is the major cause of severe acute lower respiratory tract infection in infants, the elderly population, and immunocompromised individuals. There is still no approved vaccine or antiviral treatment against RSV disease, but new monoclonal prophylactic antibodies are yet to be commercialized, and clinical trials are in progress. Hence, urgent efforts are needed to develop efficient therapeutic treatments. RSV RNA synthesis comprises viral transcription and replication that are catalyzed by the large protein (L) in coordination with the phosphoprotein polymerase cofactor (P), the nucleoprotein (N), and the M2-1 transcription factor. The replication/transcription is orchestrated by the L protein, which contains three conserved enzymatic domains: the RNA-dependent RNA polymerase (RdRp), the polyribonucleotidyl transferase (PRNTase or capping), and the methyltransferase (MTase) domain. These activities are essential for the RSV replicative cycle and are thus considered as attractive targets for the development of therapeutic agents. In this review, we summarize recent findings about RSV L domains structure that highlight how the enzymatic activities of RSV L domains are interconnected, discuss the most relevant and recent antivirals developments that target the replication/transcription complex, and conclude with a perspective on identified knowledge gaps that enable new research directions.

## 1. Overview of RSV 

The human respiratory syncytial virus (RSV) infects almost all children before the age of 2 years worldwide and is the most common cause of severe acute lower respiratory tract infections (ALRTI), such as bronchiolitis and pneumonia, in children, the elderly, and immunocompromised individuals [1,2] (http://perchresults.org, accessed on 1 September 2022). The RSV was isolated in 1955 from chimpanzees suffering a respiratory illness, and, in the following years, the virus was sampled in infants with severe lower respiratory tract disease [3,4]. Globally, 101,400 RSV-attributable overall deaths were estimated in children aged 0–60 months in 2019, with more than 97% occurring in low-income and middle-income countries [5]. In addition, 14,000 global in-hospital deaths were estimated in older adults aged ≥65 years in 2015 [6].

RSV is usually transmitted by direct contact, although it can be spread by aerosolized droplets [7,8]. RSV infection can occur very early in life despite the transplacental maternal antibodies transmission before birth and during breastfeeding, and reinfection occurs throughout life, even within the same epidemic season [9,10]. Although the correlates of protection against RSV infection remain elusive, cell-mediated immunity, mucosal immunoglobulin A, and neutralizing antibodies have been associated with protection against RSV infection. As such, natural RSV infection elicits only short-lived protective immunity [10,11,12]. 

RSV infection is initiated by viral replication in the upper respiratory tract that leads to symptoms such as congestion, runny nose, fever, cough, and sore throat. The virus next disseminates to the lower respiratory tract. Thus, RSV infection may result in airway narrowing that causes bronchiolitis, or can lead to pneumonia, in young children and acute respiratory illness in the elderly or adults at high risk [10,13]. 

Currently, there is no vaccine or effective antiviral therapy against RSV, and the only pharmaceutical intervention since 1998 has been passive prophylaxis with a RSV fusion protein inhibitor monoclonal antibody (mAb) (i.e., Palivizumab). However, its use is limited to high-risk infants because of the elevated cost and moderate efficacy [14,15]. Recently, Nirsevimab, a long-acting monoclonal antibody for the prevention of RSV infections in newborns and infants, was approved by several regulatory agencies around the world [16,17,18]. RSV vaccine development has proceeded cautiously, particularly in RSV-naïve infants, due to the results of a series of clinical trials in 1966 in the US with a formalin-inactivated vaccine against RSV (FI-RSV). Aberrant immune responses to natural infection after immunization resulted in vaccine-enhanced respiratory disease (ERD), also known as antibody-dependent enhanced disease in infants [19,20]. The current vaccine development relies on the two RSV major antigens, the F and G proteins, that protrude from the surface of the viral membrane and are the only two proteins targeted by neutralizing antibodies [21,22,23]. More than 30 different vaccine candidates are in preclinical or clinical development, as well as mAbs [12]. In addition, there is a growing interest in developing small molecule inhibitors targeting the fusion process or antiviral compounds against the replication/transcription machinery.

## 2. RSV Virion and Genome

RSV is a non-segmented negative-sense (NNS), single-stranded RNA virus that belongs to the *Orthopneumovirus* genus, *Pneumoviridae* family, *Mononegavirales* order (https://ictv.global/taxonomy/, accessed on 1 September 2022). The *Mononegavirales* order includes other important human pathogens, such as rabies (RABV), Nipah (NiV), measles (MeV), mumps (MuV), and Ebola (EBOV) viruses. Within the *Pneumoviridae* family, the human metapneumovirus (hMPV) is also an important pathogen in children [24,25]. Human RSV features two major antigenic subgroups, A and B, both circulate simultaneously, with the genotype A being the predominant subgroup [26]. In addition, the *Orthopneumovirus* genus includes the bovine RSV (BRSV), which has an important economic impact on animal farms [27,28]. 

The RSV filamentous virion consists of a lipid bilayer envelope that displays the attachment (G), the fusion (F) glycoproteins, and the small hydrophobic (SH) protein. In the core, the viral genomic RNA of negative polarity coated by the nucleoprotein (N), which is tightly associated with the large polymerase subunit (L) and the phosphoprotein polymerase cofactor (P), forms the helical ribonucleotide complex (RNP) [29,30,31]. This complex is supported by the matrix (M) protein that builds an endoskeleton assembled as a helical lattice that coordinates the arrangement of envelope-associated glycoproteins, which are also found to be helically ordered [32,33]. 

The 15.2 kb RSV genome contains 10 genes encoding 11 proteins. The 3′ end of the genome codes for the NS1 and NS2 proteins that inhibit antiviral responses, including the interferon pathway (review in [34]). It is likely that NS1 also interferes with cell transcription [35,36,37]. The subsequent genes code for the N, P, M, SH, G, and F proteins, followed by the M2 gene, which has two overlapping open reading frames (ORFs) encoding the M2-1 and M2-2 proteins. M2-1 is a transcription and processivity factor that is required for the efficient transcription of viral RNA [38,39,40]. It was shown that M2-1 interacts with viral mRNA but also with some cellular mRNAs, implying that it may have an additional role in the fate of viral mRNA following transcription [41,42,43,44,45,46,47]. M2-1 colocalizes with mRNA in inclusion bodies-associated granules (IBAGs) [41]. The M2-2 protein is a regulatory factor involved in the balance between RNA replication and transcription. Compared to a wild-type virus, an RSV M2-2 ORF knockout virus was associated with a reduction in the accumulation of genomic and antigenomic RNA, as well as an increase in the accumulation of mRNA. This predicts two activities for M2–2. The first is to increase RNA replication, and the second is to reduce transcription [48]. RSV lacking M2-2 grew less efficiently than the wild-type parent in HEp-2 cells, displayed a small-plaque morphology, and its replication in the upper tracts of cotton rats was highly diminished [49]. Interestingly, the overexpression of RSV M2-2 was shown to inhibit infection by rearranging the ribonucleocapsid complex [50]. Nevertheless, the exact molecular mechanisms of M2-2 remain unknown.

## 3. Replicative Cycle of RSV

The infectious cycle of RSV begins upon attachment of the virion to the apical surface of ciliated airway epithelial cells, which is facilitated by the viral G glycoprotein via the CX3C chemokine receptor 1 (CX3CR1) or the heparan sulfate proteoglycans (HSPGs) [51,52,53,54]. Viral entry is subsequently mediated by the F glycoprotein, which is a trimeric class I fusion protein that undergoes a drastic conformational change, that drives fusion of the viral envelope with the host cell membranes [55]. RSV F has been shown to interact with nucleolin, epidermal growth factor (EGFR), insulin-like growth factor-1 receptor (IGF1R), and intercellular adhesion molecule-1 (ICAM-1) [54,56,57,58]. 

After fusion of the viral membrane with the cellular membrane, the RNP complex is released into the host cell cytoplasm, and the genome is replicated and transcribed inside cytoplasmic viral factories forming IBs [41]. These structures, whose scaffolds are made by RNA-N-P interactions, correspond to liquid–liquid phase separation compartments that bring together all components of the polymerase complex, whose concentrations (in particular that of the polymerase and template) compartmentalize and increase the enzyme activity. It is likely that IBs also incorporate some cellular proteins [29,41,45,59,60]. Such condensates could also play a role in hiding the viral replication machinery from innate immune sensors by sequestering immunostimulatory proteins [57]. Viral mRNAs, produced by a discontinuous RNA synthesis mechanism, are transiently concentrated in IBAGs before export to the cytosol for translation into proteins [41]. RSV virions are then assembled near the plasma membrane where the F and G proteins, which transit through the Golgi complex secretory pathway, are thought to recruit M proteins that initiate the budding. Finally, the virions detach and release as filamentous particles that are ≈130 nm in diameter and 0.5–12 μm in length [61,62]. 

In addition to virus production, RSV-infected cells expressing high amounts of F proteins, have been shown to fuse with neighboring cell membranes to generate large areas with multinucleated cells. This propensity to form syncytia is one of the most striking properties discovered for RSV, and it contributes to its cytopathic effect [4].

## 4. RSV L Domains: Structural Insights and Enzymatic Activities

**Figure 1:**
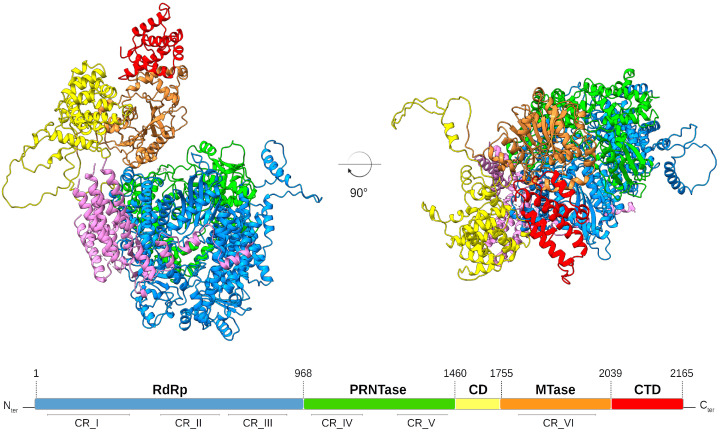
Structural model of the RSV L protein. The RSV L domains were structurally modeled and are shown as ribbons colored in: blue, RNA-dependent RNA-polymerase (RdRp) domain; green, polyribonucleotidyl-transferase (PRNTase) domain; yellow, connector domain (CD); orange, methyltransferase (MTase) domain; red, C-terminal domain (CTD); purple, tetrameric phosphoprotein aligned from the RSV polymerase complex structure (PDB: 6PZK). The linear scheme shows the RSV L protein domain organization with amino acid residue numbers indicating the functional domain boundaries. The conserved regions (CR) within the L proteins of NNS RNA viruses are indicated. RSV L structure was predicted by AlphaFold2 and interpreted with AlphaPickle [63,64]. Figures were prepared with CHIMERAX [65,66].

To ensure transcription and replication, the 250 kDa RSV L protein harbors three conserved enzymatic domains: the RNA-dependent RNA polymerase (RdRp), the polyribonucleotidyl transferase (PRNTase/capping domain), and the methyltransferase (MTase) domain, followed by the C-terminal domain, which is the most variable domain in NNS viruses (Figure 1). The RSV L protein thus possesses all enzymatic activities necessary to catalyze RNA synthesis, which form a kind of cap assembly line involved in 5′-RNA capping and its subsequent N7 and 2′-*O*-methylations, as well as the 3′ end polyadenylation of each RNA transcript. RSV RNA synthesis requires its association with the tetrameric phosphoprotein (P) to connect L with the encapsidated viral genome. It is worth noting that M2-1 is also required for efficient RNA transcription [40].

The 3.2-Å cryo-EM structure of the RSV L bound to tetrameric P has been recently resolved, revealing a striking tentacular arrangement of P, with each of the four monomers adopting a specific conformation [67,68]. In addition to the interactions between P and L, the RSV cryo-EM structure provides structural information concerning the interplay between the RdRp and the capping domains of RSV L, which exhibits that both domains are intimately intertwined. This feature shows how RNA synthesis initiation by the polymerase can be regulated by the capping domain. The structural organization of the L protein provides a framework for determining the molecular underpinnings of RSV replication and transcription and should facilitate the design of RSV inhibitors (see below).

- RSV phosphoprotein (P) cofactor

The L polymerase is always bound to its cofactor, P. The 27 kDa P protein is an essential cofactor for the RSV polymerase that ties L with the RNP complex and acts as a chaperone for the N protein by preventing the association of the neo-synthesized RNA-free N^0^ with host cell RNAs [29,30,31,69,70,71,72]. The most C-terminal residues of P bind to the genomic RNA-N (nucleocapsid) complex, thereby allowing the loading of the L–P polymerase complex to its template for replication and transcription. Thus, P plays critical roles in the regulation of RNA transcription and replication due to its interaction with multiple proteins, such as M2-1 or cellular phosphatase PP1 [38,39,40,43,73]. The P protein has been shown to present multiple sites of phosphorylation, but the exact role in its activity is still puzzling [74,75].

Structurally, RSV P contains three domains: an N-terminal domain, a central oligomerization domain forming a tetrameric coiled coil, and a C-terminal domain [67,71,76,77,78]. The N-terminal domain of unbound P is intrinsically disordered, as well as in the RSV L–P complex used for the structure resolution. The C-terminal domain was shown to display a stable cooperative structure that cannot be solved by NMR. These regions display marked dynamic heterogeneity as they adopt defined conformations when bound to other proteins [67,71,79,80]. The interaction of P with L stabilizes folded conformations of the P C-terminal region, thereby allowing them to be resolved for the first time [67,68]. RSV P forms highly stable tetramers and displays unique structural plasticity, with each monomer adopting a different conformation with distinct regions on L that also includes P–P interactions. These different conformations allow P to wrap around L in a tentacular manner, and only a relatively small region of the P tetramer reaches an extensive area on L [67]. This property is conserved among *Mononegavirales*, as the tetrameric phosphoprotein wrapping the L protein was also mapped in the N-terminal region of the hMPV or Ebola virus VP35 complex [81,82]. The parainfluenza virus 5 (PIV5) polymerase structure distinguishes the oligomerization domain of the P protein that is associated with the RdRp domain of L and protruding away from it, while the C-terminal domains of the P protein bind two discrete regions of the N-terminal region of L [83]. Conversely, for some members of the *Rhabdoviridae* family, such as vesicular stomatitis virus (VSV) and RABV, different interactions between L and P were reported [84,85,86]. In both structures, the five L domains were visible owing to a fixation of the CD, MTase, and CTD domains to the RdRp and capping domain module by a modeled segment of the N-terminal region of P. The P protein, by contacting the C-terminal region of L rather than the N-terminal region of L, as was the case in the RSV and hMPV structures, was likely to create a closed, and, therefore, less flexible, arrangement of the L domains that might represent a preinitiation or initiation compatible conformation for RNA transcription or replication.

In addition, extensive interactions between RSV P monomers were identified within and outside the oligomerization domain. Regions involved in P–P and P–L interactions were evaluated by amino acid substitutions and were shown to be critical for its function in RSV replication [67]. 

Altogether, the different structures reveal various modes of interaction between P and L that are probably necessary to allow for conformational changes of the polymerase complex that are required to ensure the initiation/elongation, transcription/replication, capping, and methylation steps. In the next paragraph, we described in more detail the structure and the function of the RSV L protein domains.

### 4.1. RNA-Dependent RNA Polymerase Domain Structure

**Figure 2:**
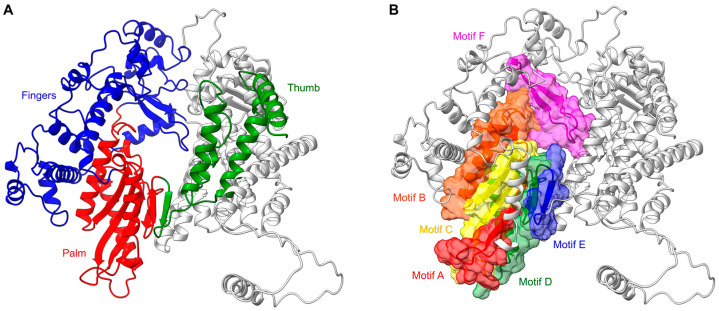
RSV RdRp features. (**A**) RdRp subdomain organization shown in ribbons with the palm in red, the fingers in blue, the thumb in green, and the remaining supporting structure in light gray. (**B**) Same position as in (**A**) with sequence motifs (A–F) of the active site shown in colored ribbons and highlighted surfaces.

**Figure 3:**
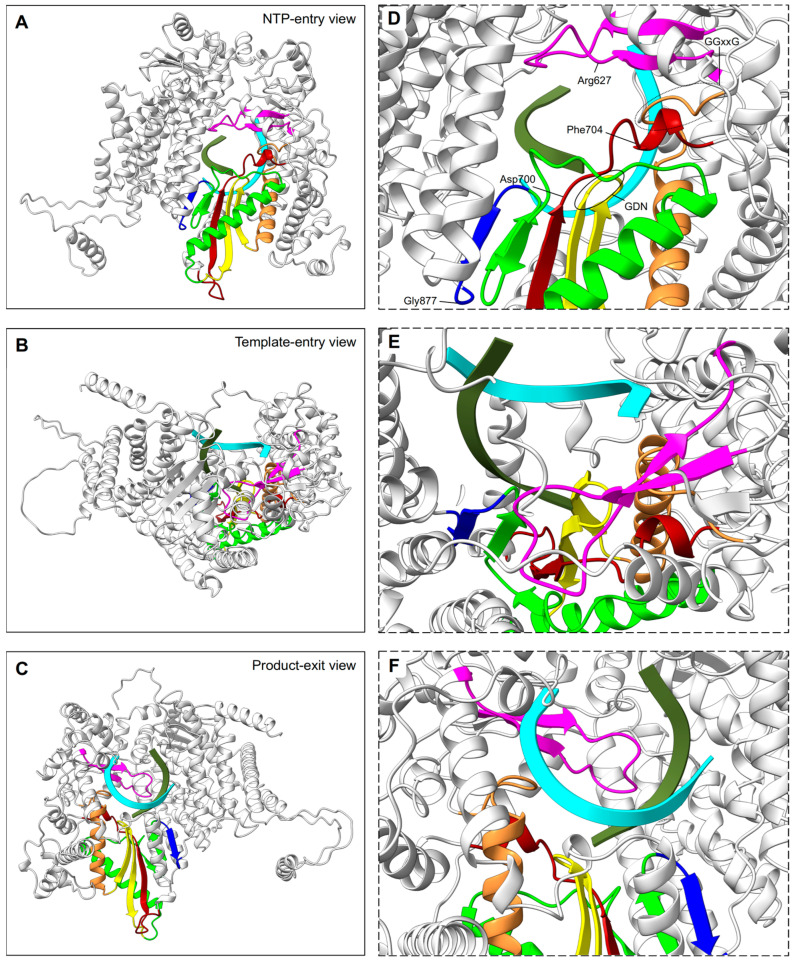
RSV RdRp channels of RNA synthesis stages. Left panels (**A**–**C**) display the RdRp domain as ribbons, viewed from the back, top, and bottom, respectively, to indicate: (**A**), NTP entry; (**B**), template entry, and (**C**), double RNA strand exit channels. Reference RdRp position and color coding of sequence motifs of the active site are the same as in Figure 2B. Right panels (**D**–**F**) are framed with a dashed line to indicate a close up of the active site from the respective left view. Aligned primer (olive) and template (cyan) molecules from the HCV polymerase structure (PDB: 4WTA) are shown in ribbons [94]. In (**D**), select residues relevant for catalysis are indicated, and conserved sequences are highlighted, including GDN (full line oval) and GGxxG (dotted line oval).

The N-terminal RdRp domain of RSV L protein displays a right-handed architecture (like all the viral RNA dependant RNA polymerases), with four subdomains: the palm, fingers, thumb, and a subdomain thought to serve as a structural support [87,88] (Figure 2A). Thus, the core of the RSV polymerase is strikingly structurally conserved with, for example, the one of the hepatitis C virus (HCV), in spite of low sequence identity (RMSD:1.3 Å vs IDseq: 4.4%). The RSV RdRp contains three conserved regions (CR_I, CR_II, and CR_III) and six conserved sequence motifs (motifs A–F), of which the majority are found in the palm subdomain that is composed of two α helices and a β sheet comprising five strands [67,87] (Figure 2B). These motifs participate in the RNA template accommodation, facilitate the incoming nucleotide, and ensure flexibility to the polymerase [89] (Figure 3). Moreover, the motif B, with the conserved glycine-rich sequence GGxxG, harbors most of the inhibitor-escape mutations, called « Quad » substitutions, and contributes to increasing the ability of the RSV RdRp to discriminate between a natural NTP and NTP analogs that are used as inhibitors [89,90]. The catalytic sequence GDN (Gly810, Asp811, Asn812) of the RSV RdRp, together with a catalytic aspartate residue (Asp700), are located in motif C (CR_III) and A, respectively. These residues coordinate the two magnesium ions required for catalysis of the phosphodiester bond formation [91]. The RSV RdRp fingers subdomain is mostly formed by α helices, but displays a small β sheet shaping the motif F [67]. This subdomain contributes to the formation of an electropositive pore that is likely serving as the entry point for incoming NTPs by interacting with the structural subdomain (CR_I), and which also has a role in coordinating the template strand [92,93].

*Mononegavirales* L proteins are structurally homologous, with the most N-terminal domain being the RdRp. The N-terminal subdomain (NTD) of the RdRp contains a clustered conserved CR_I motif, for which conserved amino acids play a part in viral transcription and, in the case of the RABV, a role in nucleocapsid engagement and template insertion has been suggested [82,86,95]. Overall, the RdRp NTD secondary structures are similar, but few insertions have been described within RdRps from viruses belonging to *Paramyxoviridae*, *Rhabdoviridae*, and *Filoviridae* without altering the fold and suggesting specific adaptation to the other partners of the replication/transcription complex (i.e., N and P) [81,82,86,95]. Structural comparison with other viral RdRps shows that *Mononegavirales*’s RdRp NTDs share structural homologies with polymerases from other viral families, including segmented negative-strand RNA and double-stranded RNA viruses [95].

### 4.2. RNA-Dependent RNA Polymerase Domain Function

**Figure 4:**
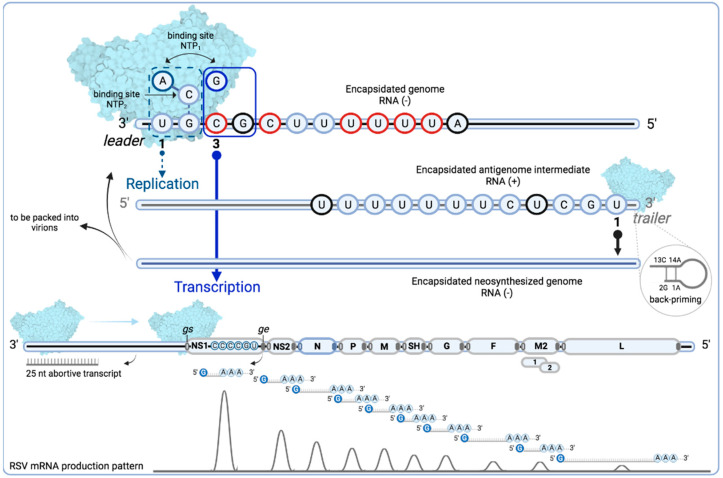
Schematic diagram of the proposed mechanisms for replication and transcription initiation for the respiratory Syncytial virus L protein. Overview of the de novo initiation at two sites in the RSV *leader* (*le*) promoter showing the two nucleotide binding sites, NTP_1_ and NTP_2_, in the catalytic center of the RdRp. The RSV polymerase complex (PDB: 6PZK) is shown in molecular surface. The first 11 nucleotides of the *le* are shown to indicate that they are sufficient to signal initiation of RNA synthesis according to a minigenome assay, with nucleotides 3, 5, 8, 9, 10, and 11 in red required for both transcription and replication; nucleotides in black show differences with respect to the *tr* sequence. The dashed line box of NTP_1_ (which has affinity for ATP or GTP) shows ATP bound to the RSV RdRp active site independently of the template sequence to prime initiation (replication in a nontemplate fashion). CTP bound to NTP_2_ contacts the GTP of the *le* promoter and makes a hydrogen bond facilitating or enabling the polymerase to catalyze phosphodiester bond formation between the ATP and CTP residues. Alternatively, the CTP could first engage the RdRp active site, followed by recruitment of ATP. This leads to pairing the ATP in the position 1U of *le* and the start of replication. The polymerase moves forward along the *le* template, adding complementary bases from 5′ to 3′ direction to produce a positive sense antigenome intermediate RNA that is encapsidated (blue oval throughout the sequence line) as it is synthesized. For production of RSV genome to be packaged in the virions, or to be used as a template for new replication/transcription cycles, the polymerase engages the position 1U of the 3′ end *tr* promoter. The width of arrows from position 1U and 3C of the *le* promoter approximately represents the relative levels of initiation from each site. The full line box of NTP_1_ shows a GTP bound to the RSV RdRp active site that will pair with the 3C position, due to the contact of CTP with the GTP on the *le* promoter, leading to transcription initiation. The RSV polymerase starts synthesizing the complementary strand that is not encapsidated and which is released after approximately 25 nucleotides, allowing the polymerase to engage in transcription and scanning for the first *gene start* (*gs*) signal (empty oval). The RSV genome is shown approximately to scale for the A2 strain, except for the NS1 gene, which is usually of the same size as NS2, but is augmented in the scheme to show the start sequence of the first RSV gene. The nascent RNA is co-transcriptionally modified with a cap structure that is added at the 5′ end by the RSV PRNTase domain. Once the polymerase reaches the *gene end* (*ge*) signal (full oval), a poly-A tail is added at the 3′ end, the mRNA is released, and the polymerase continues scanning up to the next *gs* signal. The 10 different mRNAs that encode for 11 proteins are depicted approximately to scale. M2 mRNA contains two overlapping translational open reading frames (ORFs). The curves below schematize the general, but non-linear, decline patterns of RSV mRNA production, as explained in the main text [102,103,104,105]. Created with BioRender.com accessed on 1 September 2022.

Once RSV enters a cell, the helical ribonucleoprotein complex is released into the cytoplasm, which triggers the RNA-dependent RNA polymerase (EC 2.7.7.48) activity. The RSV polymerase complex first catalyzes the transcription of viral mRNA—using the RSV genome as a template—to produce mRNAs coding for the different viral proteins (Figure 4). The replication process starts later and leads to the production of the antigenome that, in turn, will serve as a template to synthesize new viral RNA genomes. RSV presents at the 3′ ends of the genome and antigenome, extragenic regions with promoter sequences called the leader (*le*) and the trailer (*tr*), respectively, that regulate RNA synthesis [96,97,98,99]. The discontinuous mRNA transcription is controlled by a *gene start* (*gs*) signal that is present in the 5′ of each RSV gene in order to initiate mRNA synthesis. At the end of each gene, a *gene end* (*ge*) signal allows the polymerase to polyadenylate mRNA, thanks to a slipping mechanism on a uridine tract [100,101]. 

RNA synthesis by the RSV polymerase is thus a complex process, as the initiation of transcription and replication occurs at different sites (1U and 3C) that are present in a conserved promoter (*le* 3′ UGC GCU UUU UUA CGC). Initiation at two sites of the *le* promoter is performed by a de novo mechanism, due to the polymerase innate affinity for ATP and GTP [106]. The first 11 nucleotides of the *le* were shown to be necessary to initiate RNA synthesis in a minigenome assay, with nucleotides 3, 5, 8, 9, 10, and 11 being required for both transcription and replication. The saturation mutagenesis of nucleotides at positions 3, 5, 8, 9, 10, or 11 of the RSV leader sequence strongly inhibits both RNA replication and transcription in a similar manner, which reduces the detected RNA levels to less than 30% when compared to the wild type [97]. Moreover, the finding that the insertion of nucleotides 1 to 11 of the RSV leader sequence to the 3′ end of an inactive minigenome restored both transcription and replication activity confirms that these nucleotides are involved in both processes [107]. Yet, one report showed that RSV RNA polymerase activity could be performed in vitro de novo using a template of eight nucleotides, with nucleotides 3, 5, and 8 identified as essential for catalysis [108]. Of note, nucleotides 3, 5, 8, 9, 10, and 11 are similar to the RSV L *gs* signal (CCC UGU UUU A) [109]. Promoter sequences of genomes and antigenomes of viruses from *Pneumoviridae* and *Paramyxoviridae* families revealed a conserved 5′ AC sequence, which suggests that the mechanism by which the polymerases preferentially select ATP and initiate RNA replication, as described for RSV, may be relevant across families [107] (Table 1). 

For transcription initiation, the RSV polymerase engages the 3′ end *le* promoter at position 3, and first transcribes a short, uncapped RNA transcript of approximately 25 nucleotides [106,110]. After releasing this product, the polymerase remains attached to the template and scans for the *gs* signal of the first gene to reinitiate RNA synthesis. As transcription progresses towards the 5′ end, the N protein is transiently displaced from the genome template in order to allow the RSV RdRp active site to engage the RNA. Shortly after reinitiation of the RNA synthesis, the polymerase is unprocessive, the guanosine cap is co-transcriptionally added by the PRNTase domain (see below), and the RNA elongation continues, in a fully processive mode, until the polymerase reaches the *ge* signal that initiates the addition of the poly A sequence. For the transcription elongation process, particularly for mRNAs longer than ≈500 nucleotides, the RSV M2-1 protein is an essential cofactor [40,73,111,112]. The synthesized mRNA is released, and the polymerase can then transcribe the downstream gene. The hitherto widely accepted transcription model for NNS viruses thus involves sequential transcription from the 3′ promoter and transcriptional attenuation at gene junctions to generate a gradient of gene transcription that extends across the genome, with the highest level of mRNA coming from the most promoter-proximal gene, which corresponds to the NS1 gene in the case of RSV, and mRNA levels from subsequent genes drop until reaching a minimum at the most promoter-distal gene, i.e., the L gene [102,103]. However, recent reports revealed a general, but non-linear, decline in gene transcript abundance across the viral genome [104,105].

RSV genome replication implies a different process with respect to transcription. For replication initiation, the RSV polymerase engages the 3′ end *le* promoter in a template independent manner by binding ATP and initiating at position 1 [106,107]. While the replicative RNA is synthesized, it is concomitantly encapsidated by newly synthesized N proteins so that each N molecule binds seven nucleotides [113]. Encapsidation is likely to induce the polymerase to be processive and bypass the *gs* and *ge* signals [114]. This process allows for the production of the full-length antigenome, which is a (non-coding) positive stranded RNA. This replicative intermediate will next serve as a template for further rounds of genome synthesis that initiate when the polymerase engages the *tr* promoter sequence located at the 3′ end of the antigenome. The synthesized encapsidated genomes are subsequently packaged in the newly produced virions. Thus, encapsidation of the viral RNA by N proteins plays a key role in distinguishing transcription and replication. Whereas replication depends on the availability of N, specifically monomeric, RNA-free N^0^, which is achieved by P-mediated stabilization, there is no evidence that N directly induces the switch between transcription and replication [70,71,72,115]. Instead, it has been proposed that variation in NTP concentrations in infected cells governs the polymerase between transcription and replication [109]. Nevertheless, the viral machinery is a complex system, which surely does not have only one molecular switch and is susceptible to temporality for both processes (transcription and replication). 

The synthesis of the positive-sense antigenome and mRNA appeared to occur at a fixed ratio, with mRNA being by far the more abundant product [115]. How the virus ensures the specific encapsidation of its own viral genomic and antigenomic RNAs remains unknown. It was initially suggested that the affinity of N for RNA was related to sequences identified specifically in the first 35 nucleotides of the *le* promoter (i.e., 5′pppApC), which interact with the N–P complex to initiate encapsidation, and that equivalent sequences were not present in the RNA initiated at position 3 of the *le* promoter [114,116]. However, recent studies show that the nature of the 5′ end of RSV RNA does not explain the specificity of encapsidation and revealed that RNA length seems to be a key factor for stable encapsidation [60]. Indeed, both concepts provide some truth. Moreover, the fact that replication and transcription are carried out in the inclusion bodies must also play a role for the specificity of RNA encapsidation by N.

In vitro observations have also shown that the RSV polymerase can use a back-priming mechanism, which may also involve RSV replication at the *tr* promoter (*tr* 3′ UGC UCU UUU UUU CAC) to initiate RNA synthesis. In the back-priming initiation, the 3′ end of the RNA (2G and 1U) forms a hairpin structure by interacting with an internal sequence (13C and 14A) that is used as a template for the RdRp to add nucleotides to the 3′ end. In addition, nucleotides 1 and 2 can base pair with nucleotides 15 and 16 to prime the addition of CTP to the 3′ end of the RNA template [91]. The 3′ terminal extensions of 1–3 nucleotides that could be added by a back-priming mechanism have also been observed in antigenomes extracted from RSV-infected cells. Their role could be to inhibit antigenome promoter activity [117].

### 4.3. RSV Polyribonucleotidyl Transferase (PRNTase) or Capping Domain Structure

**Figure 5:**
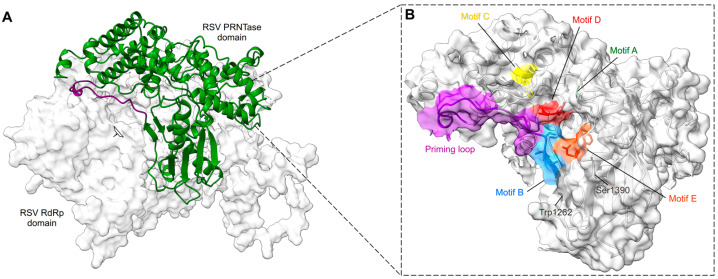
RSV PRNTase features. (**A**) The PRNTase domain is shown in green ribbons and the RdRp domain in light gray surface. The structural model of the priming loop, in dark violet, shows its position flipped up, away from the central cavity (indicated with the window) formed between the RdRp and the PRNTase. (**B**) Magnified view of the PRNTase domain with the same position as in (**A**) but shown as light gray ribbons with a transparent surface and conserved sequence motifs shown as sticks and highlighted in unique colors. The priming loop is in dark violet. Selected residues may help to form the PRNTase active site, thus anchoring motif B and D.

**Figure 6:**
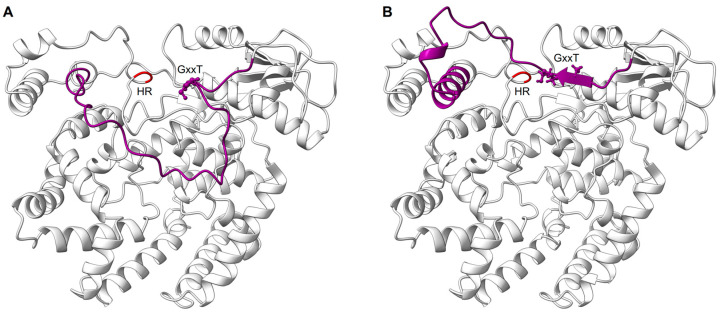
Snapshots of the PRNTase priming loop in initiation and elongation-compatible positions. The PRNTase domain is shown as light gray ribbons with the catalytic HR residues colored in red. The priming loop is shown in dark violet with the GxxT motif highlighted as spheres. The panels show snapshots of the priming loop in two different conformations—(**A**), down and (**B**), up—with respect to the RdRp domain as defined by the box in Figure 5A. This conformational change is presented in Video S1 evidencing the rearrangement undergone by the PRNTase domain. The priming loop in the down position is associated with an initiation compatible mode of the L protein for RNA synthesis. In this state, the priming loop is in the central cavity formed between the RdRp and the PRNTase domains, and it is close to the active site of the polymerase. The down conformation was modeled by alignment of the priming loop from the VSV polymerase complex structure (PDB: 5A22) to the AlphaFold RSV L model. The priming loop in the up position, as obtained from the AlphaFold RSV L model, is associated with an elongation compatible mode of the L protein and is accommodated close to the PRNTase active site, as is depicted in Figure 5A.

The RdRp domain is followed by the PRNTase/capping domain, which is responsible for the addition of a 5′ cap to nascent viral mRNAs [118]. The PRNTase domain contains the conserved regions -CR_IV and CR_V- with the conserved motifs -A to E- [119,120] (Figure 5). The cryo-EM structure of RSV L–P complex locates the PRNTase domain across from the RdRp domain (shell-like arrangement) in the same position it occupies in the VSV, hMPV, RABV, PIV5, and EBOV structures [67,81,82,83,84,85,86]. The structure of the RSV L–P complex mapped these different motifs to be clustered around the center of the cavity formed between the RdRp and PRNTase domains [67]. Two motifs, B and D, form the catalytic pocket of the capping domain. Motif D consists of the catalytic HR sequence (His1338, Arg1339), which is critical for cap formation. The histidine is involved in the transient covalent binding with the nascent RNA 5′ end. Motif B (RSV 1267–1282) contains the GxxT sequence that might accommodate the guanosine of the cap structure [67,84,118]. In some conformation, the motif B forms a loop that protrudes inside the catalytic domain of the RdRp in a position mimicking the priming loops of other RNA polymerases (i.e., VSV, RABV) that are able to ensure de novo RNA synthesis. It was thus suggested that this priming loop plays a key role in de novo initiation [84,121]. The structure of the RSV L–P complex also reveals that this loop can accommodate different positions, thanks to a glycine (Gly1264) positioned at the hinge of the structure (Figure 6 and Video S1). This hypothesis is also supported by alanine mutagenesis that shows that RSV mutants were less efficient in replication initiation, elongation, and cap addition on viral RNA [118]. 

The interplay between the RdRp and the PRNTase domain is now further supported by the comparative analysis of different snapshots of L protein from different *Mononegavirales* corresponding to (1) the initiation of transcription, (2) the elongation of the nascent RNA, and (3) the capping of the nascent RNA. All together, these data suggest that the PRNTase priming loop undergoes temporal conformational changes that regulate initiation, elongation, and mRNA capping during RNA synthesis [118,122].

### 4.4. RSV Polyribonucleotidyl Transferase (PRNTase) or Capping Domain Function

The capping of mRNA 5′ ends by the polyribonucleotidyl transferase enzyme (EC 2.7.7.8) is one important co-transcriptional RNA modification occurring during viral mRNA synthesis. The capping activity was elucidated for VSV, which proceeds by an unconventional capping pathway, and the RSV capping is supposed to proceed in a similar manner [123,124,125]. Briefly, when the nascent mRNA synthesized by the RdRp domain has reached a length of approximately 25 nucleotides, the 5′ end forms a covalent adduct with the catalytic histidine of the PRNTase domain (PRNTase-pNp-RNA) by releasing pyrophosphate. The PRNTase then transfers the RNA molecule to a GDP to synthesize the 5′ cap structure (GpppNp-RNA). It is noteworthy that the L proteins of NNS possess intrinsic specificity to catalyze capping reactions on mRNA, with a particular start sequence that is conserved among viral families [123]. After cap addition, the polymerase continues the efficient mRNA transcription (reviewed in [126,127,128]). The cap structure could be further methylated at the N7 position of the guanosine, to form the cap-0 structure (7-methyl- guanosine (m^7^Gppp)), and at the 2′-*O* position of the first and second nucleotide residues of the RNA chain to form the cap-1 (m^7^GpppNm_2′-*O*_) and the cap-2 (m^7^GpppNm_2′-*O*_Nm_2′-*O*_) structures, respectively. 

Even though the capping pathway is unconventional, the neo-synthesized cap structure is indistinguishable from cellular mRNA cap structures. Consequently, the viral mRNA escapes innate immunity, as it avoids detection as « non-self » [121,129,130,131,132]. 

As mentioned above, besides the role in mRNA capping, the PRNTase domain is involved in RNA synthesis initiation—owing to the priming loop—and modulation of the elongation properties of the polymerase during the initial steps of replication and transcription. These dual properties are distinct, as they could be uncoupled by single amino acid substitutions [118]. RNA synthesis regulation by the PRNTase may be a common feature of NNS viruses, and it is an attractive antiviral target [84,133].

### 4.5. RSV Conector Domain

The domain downstream of the PRNTase domain of the RSV L protein is the connector domain (CD) (1460–1754). A structure of the RSV CD-MTase-CTD module has not yet been elucidated, but insights can be obtained from other *Mononegavirales*. The CD has no known catalytic function; rather, it essentially plays an organizational role by positioning or spacing the catalytic domains of the RdRp from the MTase domain [84,134]. CD sequence conservation among *Mononegavirales* is weak, although common secondary structure motifs are described in VSV, RABV, and PIV5 polymerase complexes. This domain generally consists of eight α helices and long flexible linkers at each end that connect it to the PRNTase and MTase domains [83,84,85,86,135]. Small grouped basic residues located at these ends in RABV L, which are also present in VSV L, probably help guide the nascent transcript toward the capping active site and direct the capped mRNAs into the MTase active site.

### 4.6. RSV Methyltransferase (MTase–CTD) Domain Structural Insights

**Figure 7:**
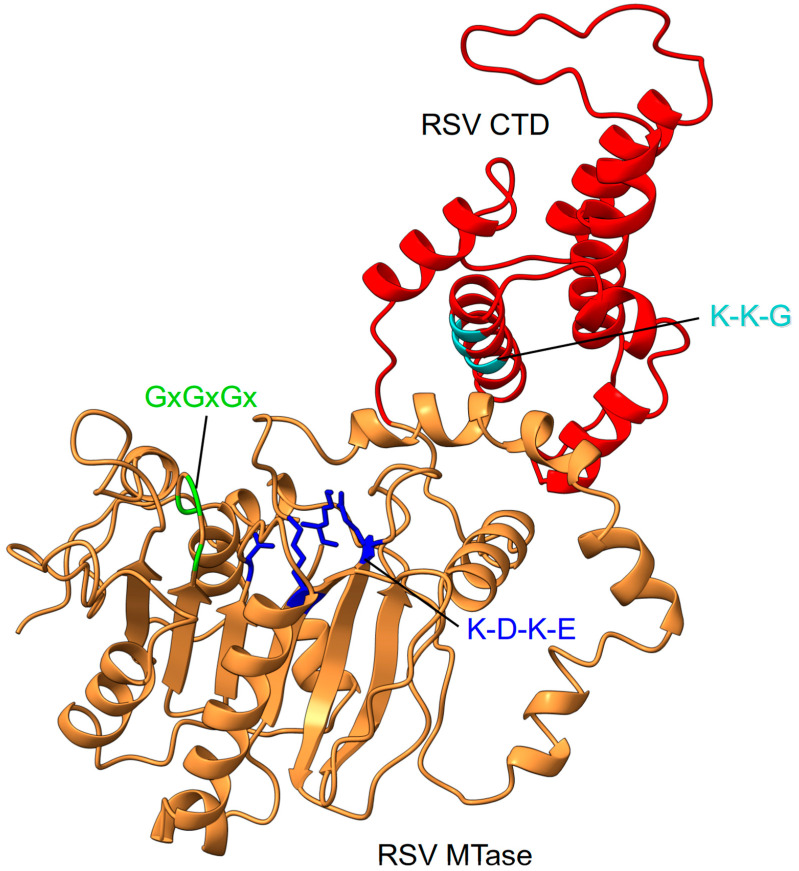
RSV MTase–CTD features. The RSV MTase is shown in orange ribbons and the CTD domain in red. Conserved sequence motifs on both domains are highlighted. The MTase catalytic tetrad is shown as sticks.

Although the structure of the RSV MTase and CTD is not yet experimentally characterized, a model can be generated that derives from the crystal structures of hMPV and the Sudan Ebola virus (SUDV) MTases that have been previously reported and provide some insights into the organization of these domains [136,137] (Figure 7). 

From a structural point of view, most of the MTases share a common structural core (Rossmann fold) made of a seven-stranded β sheet with a central topological switch point and a characteristic reversed β hairpin at the carboxyl end of the sheet (6 ↑ 7 ↓ 5 ↑ 4 ↑ 1 ↑ 2 ↑ 3 ↑). This sheet is flanked by three α helices to form a doubly wound open αβα sandwich, and is henceforth referred to as the Class I MTase structure [138,139]. The first β strand typically ends in a GxGxG motif that is the hallmark of a nucleotide-binding site, which bends sharply underneath the *S*-adenosyl-l-methionine (AdoMet) to initiate the first α helix. The only other strongly conserved position is an acidic residue at the end of β2 that forms hydrogen bonds to both hydroxyls of the AdoMet ribose.

The hMPV MTase displays the canonical Rossmann fold with some deviations, including the presence of an unusual nucleoside binding pocket adjacent to the SAM binding site. The structure lacks an obvious cap-binding site, but the protein catalyzes the methylation of the cap at its 2′-*O* and N7 positions and also efficiently methylates uncapped RNAs [136]. 

The CTD downstream of the MTase domain was shown to regulate the different enzymatic activities of the MTase domain [137]. In relation to this, the hMPV CTD is located juxtapositioned to the catalytic domain of the MTase and extends the pocket that accommodates the RNA [136]. The structure of different CTD of the L protein reveals that it forms, together with the MTase domain, a RNA-binding groove that is enriched in basic amino acids and close to the catalytic pocket. Even though this domain is quite divergent between viruses, the CTD adopts an α-helix bundle structure and sometimes a β-sheet motif, as described so far for VSV L [84]. In addition, the CTD shows some flexibility and can adopt open or closed conformations, as is shown in the RABV L. This clamp-like characteristic is thought to contribute to the repositioning of RNA needed for the subsequent N7 and 2′-*O* methylation [86]. N7-methyl transfer is thought to be promoted by optimal positioning of the reacting groups, mediated by several aromatic residues, and also by an electrostatic environment that is favorable for the enzymatic reaction [140,141]. In contrast, 2′-*O* MTases rely on the conserved catalytic tetrad, Lys-Asp-Lys-Glu [142,143,144]. 

In the structure of the RSV polymerase complex, the CD, MTase, and CTD of the L protein were not visible in the cryo-EM map, which suggests that this part of the protein can adopt different positions [67]. Among *Mononegavirales*, the structure of the VSV L–P complex forms a kind of RNA cap assembly line at an initiation competent state (probably for replication) where the three domains—CD, PRNTase, and the MTase-CTD—undergo various degrees of association with respect to the RdRp for optimal positioning for transcription or replication. The hMPV polymerase structure also suggests the flexibility of the C-terminal domains of the L protein, which may propose a possible conservation of interactions within the *Pneumoviridae* family [81]. Conversely, the RABV polymerase complex shows a compact polymerase conformation where segments of P stabilize the CD, MTase, and CTD to the RdRp and capping domains of the L. This closed conformation appears to represent the L protein positioned for initiation [86]. In the PIV5 structure, the CD, MTase, and CTD domains revealed a unique configuration, positioned directly on top of the PRNTase domain, that accommodated the active site for methylation and directly adjacent to the capping domain. This conformation would provide accessibility to the active site for capping, followed by the methylation. The repositioning of the MTase domain seems to be facilitated by the flexible linker region between the CD and the MTase [83]. 

From these observations, it can be inferred that the L protein of *Mononegavirales* is organized in a practical way where the two modules, RdRp-PRNTase and CD-MTase-CTD, are associated with each other and adopt transitory configurations for initiation, capping, and elongation.

### 4.7. RSV Methyltransferase (MTase–CTD) Domain Function

The methylation of the RNA cap structure is a post-transcriptional modification that is essential for virus replication. The canonical cap methylation mechanism implies that, after cap addition, the methylation of the guanosine moiety is catalyzed by a (guanine-N7)-methyltransferase (N7MTase), thus providing the minimal RNA cap chemical structure, named cap-0 (m^7^GpppNp), which is required for RNA translation into proteins [145,146,147]. Further methylation can target the 2′-OH of the ribose from the first and second nucleotides to yield cap-1 (m^7^GpppNm_2′-*O*_), and cap-2 (m^7^GpppNm_2′-*O*_Nm_2′-*O*_) structures, respectively. The 2′-*O*-methylation has been shown to hide viral RNA from innate immunity detection [130,131,132,148]. 

The MTase activity of the RSV MTase–CTD domain (amino acids 1755 to 2165) has been recently characterized [149]. The RSV MTase sequence contains the conserved K-D-K-E catalytic tetrad (K1831, D1936, K1973, E2004) and the SAM/SAH binding GxGxGx motif (G1853, G1855, G1857), followed by the K-K-G motif (K2149, K2153, G2156) within the CTD, which is required for RNA substrate binding. Comparison of the enzymatic activities of the MTase expressed as a single domain, or in the context of the full-length L protein, demonstrated that both purified recombinant proteins methylate the N7 and 2′-*O* positions of capped synthetic RNAs that mimic the RSV mRNA 5′ end. Thus, the biochemical data suggest that the MTase domain has evolved to accommodate RNA in different positions in order to ensure both N7 MTase and 2′-*O* MTase activities by using one single SAM-binding site [150]. In contrast to the SUDV MTase that catalyzed the guanosine N7 methylation and catalyzed unexpectedly high levels of internal adenosine 2′-*O* methylation (as the enzyme is also able to methylate uncapped RNAs), the RSV MTase did not induce internal methylation, which suggests that it is a strict cap-dependent MTase [149,151]. Accordingly, the cap or RNA recognition mechanism might be specific for the different NNS virus MTases that share a conserved MTase domain, but show striking differences in the CTD that differ in size, sequence, and that lack any conserved signature [142]. Nevertheless, the CTD plays a key role in RNA binding and, consequently, in regulating the different MTase activities [136,137]. The role of the CTD was recently highlighted for the SUDV MTase that lost its RNA binding properties and enzymatic activity in the absence of the CTD [137]. 

The biochemical studies also give interesting information regarding the sequence of cap methylation. For RSV, it is likely that the N7 methylation occurs before the 2′-*O*-methylation, as time-course methylation reactions using the Gppp GGG ACA AAA (RSV_9_) substrate revealed ^m^GpppG-RSV_9_ as the first product, with an initial velocity that was about 12-fold faster than GpppG_m_-RSV_9_ synthesis [149]. This order of methylation is different from most mononegaviruses (VSV, hMPV, Ebola virus) in which 2′-*O*-methylation was proposed to precede N7 methylation [123,136,151,152,153].

## 5. RSV L Antivirals

**Figure 8:**
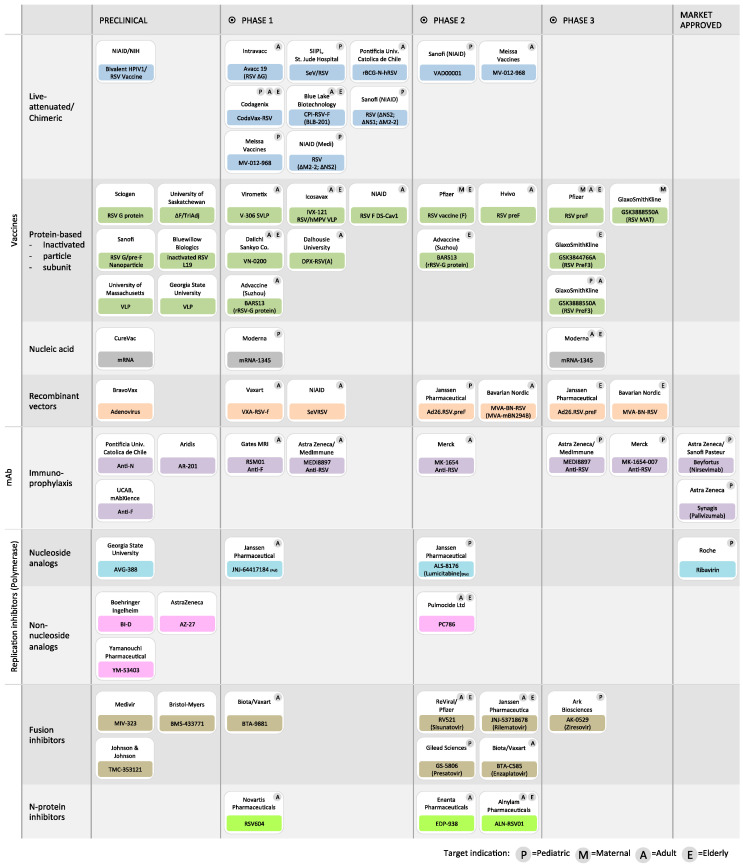
Snapshot of the development of RSV clinical interventions. Adapted from the PATH website at www.path.org (accessed date 9 January 2023) and [155,156,157,158,159,160,161,162,163,164].

Therapeutic opportunities to address the ongoing burden of RSV disease are currently progressing via different strategic approaches, including the development of mAbs, small molecules, and vaccines. However, each strategy encounters its own challenges and, even if some molecules show potential in vitro, not all advance to clinical development, due to pharmacokinetic properties or safety factors [154]. In this review, we focus on the development of antiviral strategies targeting the L protein, which orchestrate the replication and transcription, and we will only describe the most relevant and recent molecules. Exhaustive references of antivirals and a snapshot of the clinical interventions targeting RSV have been reviewed in www.path.org (accessed date 9 January 2023) and [155,156,157,158,159,160,161,162,163,164] (Figure 8). The current approaches to inhibiting the RSV polymerase include nucleoside and non-nucleoside analogs inhibitors that have been identified by screening compounds using infectious RSV, cell-based replicon assays, and in vitro assays that use the recombinant L–P complex and naked RNA as templates. 

### 5.1. Nucleoside Analogs

Ribavirin is the only FDA approved antiviral treatment for RSV. It is a guanosine analog and broad-spectrum antiviral drug that inhibits the replication of both RNA and DNA viruses by direct (interference with RNA capping, polymerase inhibition, lethal mutagenesis) and indirect (inosine monophosphate dehydrogenase inhibition and immunomodulatory effects) mechanisms [165,166]. Ribavirin can be administered intravenously, orally, and by aerosolization. It was approved by the FDA in 1986 for the aerosol treatment of serious RSV infections in hospitalized children [167]. Nevertheless, its use has been controversial because of the potential teratogenicity of inhaled ribavirin particles and its high cost [168]. Yet, oral ribavirin and corticosteroid have been well-tolerated and cost-effective therapeutic regimen in the treatment of lung and heart/lung transplant recipients [169]. A phase 4 trial showed a higher efficacy in preventing RSV ALRTIs in high-risk patients when using an intermittent rather than a continuous dosing schedule [170].

A screening for the discovery of new scaffolds of nucleoside analogs for RSV inhibitors led to the identification of a 2′difluoro-4′azido-cytidine, which is an analog of gemcitabine that is also known to inhibit HCV polymerase. Modifications at the 2′ and 4′ positions tended to improve anti-RSV potency and selectivity, have led to the design, synthesis, and pharmacokinetic assessment of a series of 4′-substituted cytidine nucleosides, from which ALS-8176 (4′-Chloromethyl-2′-deoxy-3′,5′-di-O-isobutyryl-2′-fluorocytidine), also known as JNJ-64041575 or Lumicitabine, was identified [171]. From these experiments, it was concluded that the selectivity towards RSV (and not for HCV) is provided by a combination of the 2′F- and the 4′ClCH_2_ moieties on the ribose group. On the other hand, the presence of the 2′Me-moiety led to a lack of recognition by the RSV polymerase. ALS-8176 targets the RSV polymerase and acts as a chain terminator of RNA synthesis and inhibitor of viral polymerization activity. Some resistant mutations to ALS-8176 have been identified in vitro and mapped to the « Quad » substitutions—in the motif B of the RdRp domain—in RSV L (M628L/A789V/L795I/I796V) proteins [90]. An allosteric mechanism has been proposed in which the conformation of the polymerase active site is altered, without directly contacting either native NTPs or the cytidine analog [67]. ALS-8176 possesses high oral bioavailability and has been demonstrated to be efficacious in a first clinical trial with healthy adults inoculated with RSV [172]. In phase 2a trials, the molecule showed more rapid RSV clearance and a greater reduction of viral load, with more accompanying improvements in the severity of clinical disease in treated groups than in the placebo group. Results in phase 2b trials had been placed on hold. JNJ-64417184 is another RSV polymerase inhibitor that is currently under evaluation in phase 1 trials. 

AVG-388 is a nucleoside analog from a novel AVG class of allosteric inhibitors of RSV that has been shown to effectively block the activity of the RSV polymerase in vitro by disturbing the initiation of viral RNA synthesis at the promoter. AVG-388 is orally available and showed potency in the RSV mouse model when administered therapeutically [173,174].

### 5.2. Non-Nucleoside Analogs

BI-compound D (BI-D) from the imidazoquinolines family has multiple effects on the RSV polymerase function. It inhibits full-length mRNA synthesis due to a disruption in cap addition and producing aborted transcripts (40–50 nucleotide length) with 5′ triphosphate moiety [175,176]. In addition, BI-D affects the processivity of the polymerase within the promoter region. Interestingly, higher levels of interferon and interferon-stimulated genes were detected in RSV-infected cells treated with BI-D [177]. These results suggest that molecules that also inhibit the capping activity of the RSV polymerase could have the potential to activate the innate immune response in addition to the inhibition of the polymerase. According to the structural analysis of the RSV polymerase complex, viral escape from BI-D has been related to substitutions in residues surrounding the PRNTase active site (I1381S, L1421F, or E1269D) that may indirectly alter the shape of the pocket and possibly enabling the binding of the compound [67,176].

AZ-27 is a derivative of YM-53403 from a benzazepine series that showed specific inhibition to RSV in vitro [178]. The compound inhibits the initiation of de novo RNA synthesis at the promoter, which results in the inhibition of both mRNA transcription and genome replication [179]. The mechanism of action is not clearly known, but it has been suggested to have a possible involvement in the capping and methylation domains [180].

PC786 is a nebulized non-nucleoside RSV polymerase inhibitor that is active against RSV-A and RSV-B clinical isolates. The small molecule tested in a challenge study was well tolerated and demonstrated antiviral effects. These results are proof-of-concept that a nebulized, non-nucleoside antiviral can inhibit RSV replication without causing local irritation, while limiting the potential for systemic side effects [181].

## 6. Conclusions and Outlook

We are at an exciting time in RSV research as a result of recent advances in the knowledge of the structure and function of viral proteins, the immune responses to natural infection, and the causes of ERD. This is evidenced by the landscape of prophylactic and therapeutic agents in clinical or preclinical development, expected to be efficacious owing to well supported knowledge, to ultimately initiate a global struggle against this ubiquitous human pathogen. However, much remains to be done in order to fully understand the molecular mechanisms of RNA replication and transcription. Structural resolution of the different intermediaries of RSV RNA transcription and replication initiation/elongation reactions is not yet available (i.e., structures of the RSV L–P complex with a primer-RNA template or RNA template alone). In addition, structural information on the RSV CD-MTase-CTD module will enhance our understanding of the nature and degree of association of the RSV L domains and may contribute to the design and evaluation of antivirals that target the RSV MTase activity. Additionally, the structural resolution of an RSV L–P complex bound to the nucleocapside template will allow us to understand how the L–P complex accesses the 3′extremity of the genomic RNA.

Reliable enzymatic assays for PRNTase activity needed to develop antivirals that are specific for these functions are lacking. Future experiments should demonstrate the RSV L capping activity and provide a decorticate description of the RSV PRNTase activity by (1) analyzing the covalent intermediate complex between the PRNTase domain of the RSV L protein and the RNA (L-pRNA), showing the RNA sequence specificity and (2) demonstrating the pRNA transfer to a GDP molecule acceptor as a proof-of concept of the RSV capping reaction.

In addition, evaluation of the action of antivirals in acute infection and in children with bronchiolitis remains an open question. This aspect also demands the structural resolution of the RSV L–P complex bound to antivirals. 

Given the fact that RSV has evolved a sophisticated set of viral proteins and mechanisms for escaping host immune components during infection, as an optimized machinery for accurate transcription and replication, the development of effective therapeutic agents and vaccines has been significantly challenging. Therefore, a complete understanding of the clinical and immunologic aspects of RSV infection is still needed. 

RSV possesses different well characterized enzymatic activities within the L protein, which are required for successful viral replication and, therefore, represent appealing targets. The rational design of blocking molecules is becoming increasingly plausible. Moreover, the evaluation of molecule combinations may reveal a synergistic enhancement of inhibitory effects that can apply as alternative therapy to prevent drug resistance.

## 7. Materials and Methods

- RSV L protein modeling

Structural predictions were conducted on the L protein of RSV (UNIPROT: G8EJ12_HRSV). We used a local installation of AlphaFold2 to perform the full-length prediction (2165 residues) [63]. Given that RSV L is a multidomain protein of which only the polymerase and PRNTase core have been experimentally solved by cryo-EM in complex with its co-factor P (PDB: 6PZK), and it is known to undergo conformational changes, we decided to use a naïve prediction protocol (without PDB input) and use the experimental structure as external validation. Since the different domains are separated by a long flexible region, we used the core structure as a filter to select the most likely compatible positions of the CD, MTase, and CTD domains (i.e., no steric clash between domains). The reliability analysis of the model prediction was done using AlphaPickle, and the pLDDT values were outputted in the B-factor field of the PDB file for each prediction [64]. Structure representations were performed with ChimeraX [65,66].

- Modeling of the PRNTase priming loop conformational change:

The priming loop in the up position was obtained from the AlphaFold RSV L model. The homologous structure of the VSV L (PDB: 5A22) has its priming loop in a down conformation that blocks the exit path to RNA. The polymerase-PRNTase domains of VSV present an overall root mean square deviation (RMSD ~1, 3 Å) with the ones of RSV. The major distortion was observed around the priming loop, which makes the VSV structure a good template for modeling the loop. Using MODELLER and a templated structural alignment between VSV and RSV, a model of RSV with the priming loop in a down conformation with the best discrete optimized protein energy (DOPE) score was generated and selected [182,183].

## Figures and Tables

**Table 1:** Alignment of the 5′ genome sequence of viruses for the *Pneumoviridae* and *Paramyxoviridae* families. The conserved sequence (AC) is shown in red.

***Pneumoviridae*** **family**	**5′ genome sequence**	**Reference**
RSV	ACGCGAAAAAA	NC_001803.1
Bovine RSV	ACGCGAAAAAA	NC_001989.1
Pneumonia virus of mice	ACGCGAAAAAA	NC_006579.1
Human metapneumovirus	ACGCGAAAAAA	FJ168779.1
Avian metapneumovirus	ACGAGAAAAAA	NC_039231.1
***Paramyxoviridae*** **family**		
Mumps virus	ACCAAGGGGAA	NC_002200.1
Sendai virus	ACCAAACAAGA	NC_001552.1
Newcastle disease virus	ACCAAACAGAG	AF309418.1
Nipah virus	ACCAAACAAGG	NC_002728.1
Measles virus	ACCAAACAAAG	NC_001498.1
Hendra virus	ACCGAACAAGG	NC_001906.3
Human Parainfluenza virus 3	ACCAAACAAGA	EU326526.1

## Data Availability

Not applicable.

## Supplementary Materials

The following supporting information can be downloaded at: https://www.mdpi.com/article/10.3390/v15020341/s1, Video S1: RSV PRNTase priming loop conformational change.
